# Supplementary material for: Massive culture-based approach for the screening of AmpC, ESBL, and carbapenemase producers from rectal swabs
Source: Microbiol Spectr. 2025 Jul 7;13(8):e00157-25. doi: 10.1128/spectrum.00157-25 (PMC12323602; doi:10.1128/spectrum.00157-25)
Supplement: Supplemental material — Fig. S1; detailed rationale and protocol for screening AmpC-, ESBL-, and carbapenemase-producing gram-negative bacilli using a massive culture-based approach. [file spectrum.00157-25-s0001.docx]

**Massive culture-based approach for the screening of AmpC-, ESBL- and carbapenemase producers from rectal swabs**

Gabriel Taddeucci-Rocha^1,2^, Victoria de Oliveira Costa^1^, Sarah Vitória Martins da Silva^1^, Jéssica Britto Gonçalves^1^, Natalia Chilinque Zambão da Silva^3,4^, Marcia Maiolino Garnica^4,6^, Renata Cristina Picão^1,6∗^

Supplementary material:

1. Figure. S1. Rationale of the massive-culture approach for detecting and differentiating between gram-negative bacilli producing the beta-lactamases AmpC, ESBL and carbapenemases.
2. Massive culture protocol for detecting gram-negative bacilli producing AmpC, ESBL and carbapenemases.


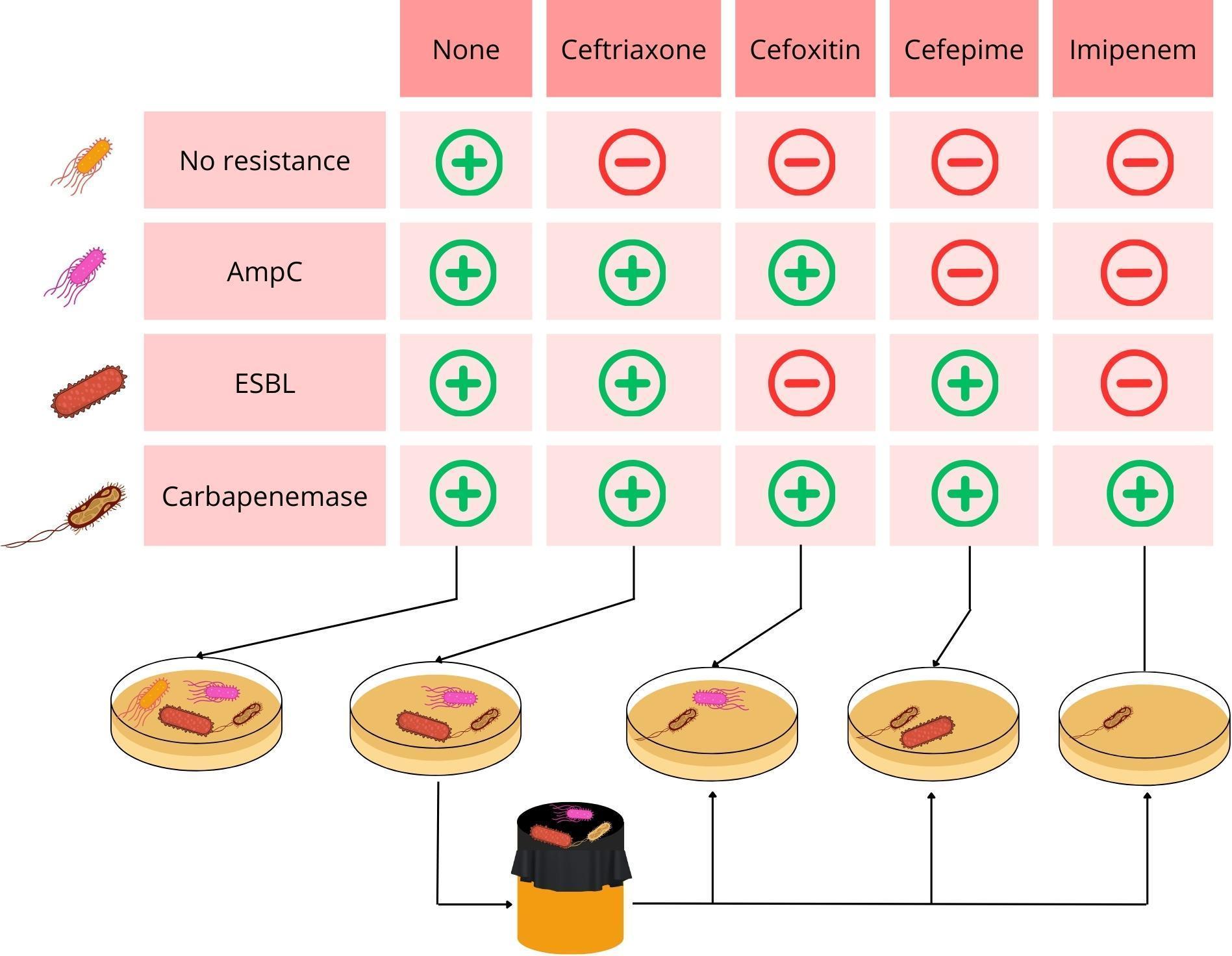


**Figure. S1**. Rationale of the massive-culture approach for the differential screening of gram-negative bacilli producing the beta-lactamases AmpC, ESBL and carbapenemases. Complex samples are seeded on MacConkey plates supplemented with ceftriaxone and after incubation, the culture is replicated on plates supplements with cefoxitin, cefepime and imipenem. Growth of all strains is expected in MacConkey plates, producing beta-lactamases or not. On plates supplemented with ceftriaxone it is expected to grow bacteria producing AmpC, ESBL and/or carbapenemases; AmpC producers are expected to grow on plates supplemented with cefoxitin, but not with cefepime and imipenem; ESBL producers are expected to grow on plates supplemented with cefepime, but not with cefoxitin and imipenem; and carbapenemases producers are expected to grow on every selective pressure.

**Massive culture protocol for the differential screening of gram-negative bacilli producing AmpC, ESBL and carbapenemases**

**Materials:**

- MacConkey agar plate for velvet sterility control
- MacConkey agar master plate supplemented with ceftriaxone (1.5 µg/ml)
- Replica plates:
  - Plate 1: MacConkey agar without antimicrobial agents (initial inoculum control)
  - Plate 2: MacConkey agar supplemented with cefoxitin (32 µg/ml)
  - Plate 3: MacConkey agar supplemented with cefepime (16 µg/ml)
  - Plate 4: MacConkey agar supplemented with imipenem (4 µg/ml)
  - Plate 5: MacConkey agar without antimicrobial agents (final inoculum control)
- Sterile 15 x 15 cm elastic velvet piece
- Replica plating support
- Laminar flow hood or Bunsen burner
- Incubator
- Plate marker

**Procedure:**

**Day 1:**

1. **Sterilize the velvet**: autoclave the velvet piece and allow it to dry in a sterile environment.
2. **Prepare the master plate**: prepare and label the MacConkey agar plate supplemented with ceftriaxone (1.5 µg/ml)
3. **Inoculate the specimen**: inoculate the specimen onto the MacConkey agar supplemented with ceftriaxone (1.5 µg/ml), and incubate overnight at 35 ± 2 ^o^C.

**Day 2**

1. **Prepare replica plates**: prepare and label the following plates:
   - Plate 1: MacConkey agar without antimicrobial agents (initial inoculum control)
   - Plate 2: MacConkey agar supplemented with cefoxitin (32 µg/ml)
   - Plate 3: MacConkey agar supplemented with cefepime (16 µg/ml)
   - Plate 4: MacConkey agar supplemented with imipenem (4 µg/ml)
   - Plate 5: MacConkey without antimicrobial agents (final inoculum control)

Arrange the plates in a logical sequence in the sterile workspace.

1. **Prepare the replicating device**: place the sterile velvet on the replica plating support.
2. **Sterility check**: stamp the velvet onto a MacConkey agar plate without antimicrobial agents to check for sterility. Gently press to ensure full contact.
3. **Stamp the master plate**: press the master plate culture onto the velvet to transfer the inoculum.
4. **Replica plating**:
   - Transfer the velvet impression onto Plate 1 (MacConkey agar without antimicrobial agents).
   - Next, transfer to Plate 2 (MacConkey agar with cefoxitin).
   - Continue transferring to Plate 3 (MacConkey agar with cefepime), then Plate 4 (MacConkey agar with imipenem).
   - Finally, transfer to Plate 5 (MacConkey agar without antimicrobial agents).
   - Ensure even pressure with each transfer.
5. **Incubation**: incubate all plates overnight at 35 ± 2 ^o^C.

**Day 3**

1. **Examine the plates**: after incubation, observe and compare colony growth each plate. Refer to Figure 2 of the manuscript for guidance.

**Interpretation of Results:**

- **Sterility control plate**: no bacterial growth should be observed.
- **Plates 1 and 5**: both should show growth of all strains from the master plate, serving as controls for the initial inoculum and throughout the replication process. Absence of colonies on Plate 5 compared to Plate 1 suggests the inoculum may have been depleted during replication on the antibiotic-supplemented plates. Therefore, the absence of resistance mechanisms on these plates should be interpreted cautiously.
- **Plate 2 (cefoxitin)**: growth suggests the presence of AmpC and/or carbapenemase-producing strains.
- **Plate 3 (cefepime)**: growth suggests the presence of ESBL and/or carbapenemase-producing strains.
- **Plate 4 (imipenem)**: growth suggests the presence of carbapenemase-producing strains.
- **Absence of growth** on any antibiotic plate suggests the absence of strains producing the corresponding resistance mechanism under that selective pressure.

**Additional Notes:**

- **The proposed approach is a screening test**, and confirmation of AmpC, ESBL and carbapenemases should be conducted with further analysis.
- **Sterility:** always work under aseptic conditions, using a laminar flow hood or Bunsen burner to minimize contamination.
- **Control plates:** the two plates without antimicrobial agents (Plates 1 and 5) are essential for validating proper transfer throughout the whole process and ensuring no contamination.
- **Velvet reuse:** after use, decontaminate the velvet by soaking it in a 2% sodium hypochlorite solution for 18-24 hours, rinse thoroughly, and then sterilize by autoclaving before re-use.
- **Stamping pressure:** apply gentle, even pressure when stamping the velvet. Excessive force can moisten the fabric too much, hindering colony observation.
- **Stamping direction:** maintain consistent stamping direction across all plates to ensure uniform colony distribution and facilitate accurate comparison.
- Refer to the supplemental video for a detailed visualization of the replication procedure.
